# Cutaneous metastasis from pancreatic cancer: A case report and systematic review of the literature

**DOI:** 10.3892/ol.2014.2610

**Published:** 2014-10-10

**Authors:** HAI-YAN ZHOU, XIAN-BAO WANG, FANG GAO, BING BU, SHU ZHANG, ZHEHAI WANG

**Affiliations:** 1 Department of Oncology, Shandong Cancer Hospital and Institute, Jinan, Shandong 250117, P.R. China; 2 Department of Thoracic Surgery, Affiliated Hospital of Jining Medical University, Jining, Shandong 272205, P.R. China

**Keywords:** pancreatic cancer, cutaneous metastasis

## Abstract

Cutaneous metastasis from pancreatic cancer is uncommon, therefore, the outcome of this progression has rarely been investigated. The aim of the present report was to evaluate the clinical characteristics of patients exhibiting cutaneous metastasis from pancreatic cancer. Thus, the current report presents a rare case of cutaneous metastatic disease from pancreatic cancer and describes a systematic review of the literature. A total of 54 articles comprising 63 cases were included for analysis. The relevant clinical and pathological characteristics, as well as the treatment strategies and survival outcomes of this rare disease presentation were reviewed. The average patient was was aged 63.9 years and males constituted a marginally greater proportion of the cohort (61.9%). The predominant manifestation of the cutaneous metastasis was a nodule or mass (73%) and the most common site of the skin lesion was non-umbilicus rather than umbilicus. The majority (66.7%) of the skin lesions were singular, particularly in patients exhibiting Sister Mary Joseph’s nodule (90%). A wide range of histological subtypes presented, with a predominance of adenocarcinoma (84.1%). Of the cases that specified the tumor differentiation grade, 78.2% were moderately or poorly differentiated. Immunohistochemistry revealed that cytokeratin (CK)20-negative, and CK7-, CK19- and carbohydrate antigen (CA)19-9-positive were specific diagnostic markers for pancreatic cancer. Distal metastases, excluding the skin, were observed in 68.3% of patients and the median survival period was 5 months. Treatment strategies including surgery, radiation, chemotherapy or a combination improved survival time from 3.0 to 8.3 months. Cutaneous metastasis from pancreatic cancer is a rare finding, often providing the only external indication of an internal malignancy and, therefore, should be considered in the differential diagnosis of skin lesions. Metastasis to the skin indicates a widespread, general dissemination and a poor prognosis. A combination of surgery, radiotherapy and chemotherapy appears to result in improved survival rates.

## Introduction

Pancreatic cancer is known to metastasize rapidly, most commonly to the liver and peritoneum, followed by the lungs, bones and brain (1,2). The occurrence of cutaneous metastases is rare, predominantly presenting in close proximity to the umbilicus and is termed Sister Mary Joseph’s nodule (SMJN) (3). Non-umbilical cutaneous metastases are rare, with only a small number of cases reported (4,5). The present report describes a case of multiple cutaneous metastatic lesions associated with pancreatic cancer, reviews the published literature regarding cutaneous metastasis from pancreatic cancer (by conducting a detailed PubMed search). Furthermore, an analysis of 63 reported cases of cutaneous metastasis from pancreatic cancer was conducted with regard to the clinical and pathological characteristics, treatment strategies and survival outcomes, thus providing an overview of this rare type of disease manifestation. Written informed consent was obtained from the patient’s family.

## Case report

**Figure 1:**
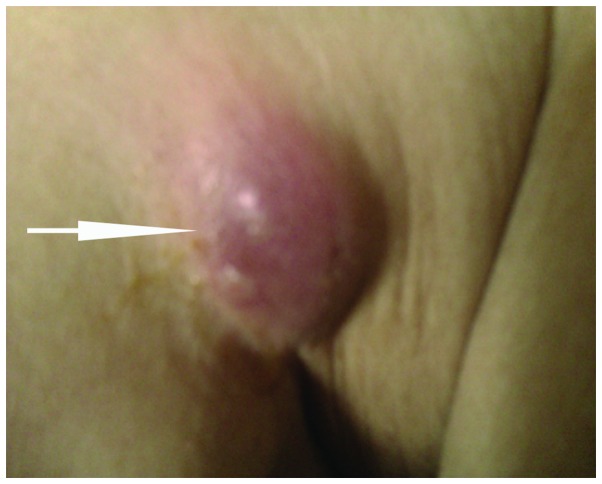
Cutaneous metastatic lesion at the right anterior axillary fold. The nodule is round, indurated and violaceous, measuring 1.5×1.5 cm.

**Figure 2:**
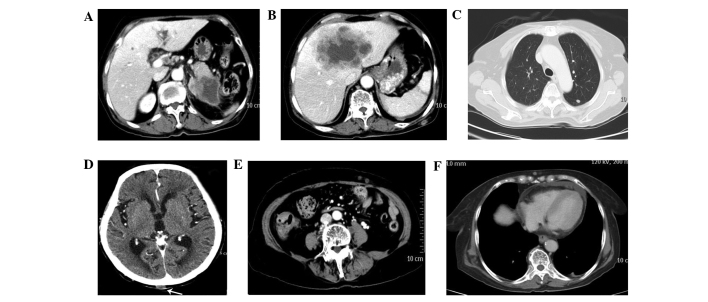
(A) Computed tomography (CT) scan revealed an enlarged pancreatic tail with a low-density soft tissue mass measuring 7 cm in diameter with (B) hepatic focal lesions and (C) lung lesions. (D) Cutaneous metastasis is present on the CT scan at the occipital scalp, (E) right upper chest and (F) left abdomen.

**Figure 3:**
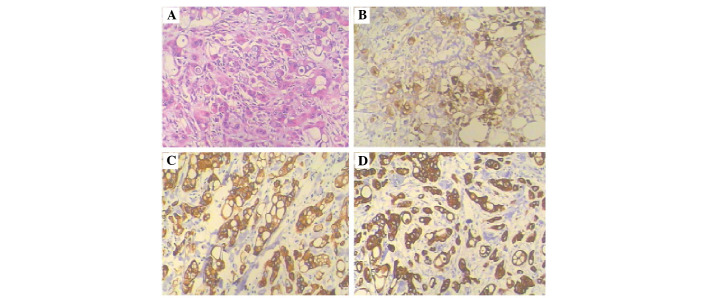
(A) Hematoxylin and eosin stain of a section from the mass of the patient’s right upper chest revealing atypical tumor cells, which have partially formed adenomatous structures or nested glandular structures (magnification, ×200). (B) Tumor cells stained positive for carcinoembryonic antigen (magnification, ×200). (C) Malignant glands are strongly positive for cytoplasmic cytokeratin 7 (magnification, ×200). (D) Tumor cells show strong cytoplasmic staining for cytoplasmic cytokeratin 19 (magnification, ×200).

In May 2012, a 76-year-old female was referred to the Department of Oncology (Shandong Cancer Hospital and Institute, Jinan, China) with the complaint of nausea and a lack of appetite for 10 days prior to referral. The patient had experienced gallstones for several years. Upon physical examination, asymptomatic violaceous nodules were observed on the right anterior axillary fold, occipital scalp, chest and abdomen (Fig. 1). Completion of the physical examination identified no further abnormalities. Routine laboratory testing revealed that renal and hepatic markers were within the normal ranges, including white blood cell (9.2×10^9^/l; normal range, 3.5–9.5×10^9^/l), platelet (301×10^9^/l; normal range, 125–350×10^9^/l) count, serum alanine aminotransferase (10 U/l; normal range, 7–40 U/l), blood urea nitrogen (3.98 mmol/l; normal range, 2.9–8.2 nmol/l), creatinine (45 μmol/l; normal range, 45–84 μmol/l) and glucose levels (5.85 mmol/l; normal range, 5.85 mmol/l). The hemoglobin (111 g/l; normal range, 115–150 g/l) levels were slightly below the normal range, potentially due to the increased age of the patient and low food uptake, the albumin (35.1 g/l; normal range, 40–55 g/l) level may be lower than the normal range due to a decrease in food uptake and decreased synthesized liver function due to metastasis, potassium levels (3.4 mmol/l; normal range, 3.5–5.3 mmol/l) were lower than the normal range potentially due to decreased food uptake and increased fibrinogen levels (4.48 g/l; normal range, 2–4 g/l) may correlate with the end-stage of the disease (6). Serum carbohydrate antigen (CA)19-9 (2.1 U/l; normal range, 0–39 U/l), cancer antigen 72-4 (5.06 U/l; normal range, 0–6.9 U/l) and α-fetoprotein (2.52 ng/ml; normal range, 0–7 ng/ml) were also within normal limits, however, carcinoembryonic antigen (CEA) was elevated to 27.54 ng/ml (reference range, 0–3.4 ng/ml). A computed tomography (CT) scan of the abdomen, chest, pelvis and brain was performed, which revealed an enlarged pancreatic tail containing a low-density soft tissue mass measuring ~7 cm in diameter. Post-peritoneum lymph node enlargement, lesions on the lungs, hepatic focal lesions (maximum size, ~8×10 cm) and multiple subcutaneous nodules were also identified on the right upper chest, occipital scalp, upper arm and abdomen (Fig. 2). Examination and thorough investigation did not detect metastases elsewhere (for example in the ovaries or brain). Abdominal ultrasound and CT findings were consistent with signs for cancer of the tail of the pancreas with multiple metastatic lesions. The nodule of skin at the right anterior axillary fold was removed for biopsy and pathological examination of the excised lesion identified a poorly differentiated metastatic adenocarcinoma involving the dermis and subcutaneous tissue. Immunohistochemical staining (Fig. 3) revealed that the tumor was weakly and focally positive for CEA (Fig. 3B) and strongly positive for cytokeratin (CK) 7 (Fig. 3C) and CK19 (Fig. 3D). Staining was negative for estrogen and progesterone receptors, thyroid transcription factor-1, and CK20 (not shown). Thus, the patient was diagnosed with stage IV disease, according to the American Joint Committee on Cancer TNM staging system for pancreatic cancer (7) and was subsequently treated with one cycle of gemcitabine. Prior to administration of the second cycle of gemcitabine (1.2 g, days 1 and 8, every three weeks), the lesion at the occipital scalp enlarged due to a superficial ulceration. As a result of disease progression, the patient was administered with oxaliplatin (100 mg, day 1, every two weeks) combined with S-1 capsules; however, the patient rapidly deteriorated and succumbed following two months of treatment. An autopsy was not permitted for this patient.

A literature search of the electronic PubMed database (www.ncbi.nlm.nih.gov/pubmed; up to July 2013) was conducted using Medical Subject Headings (www.nlm.nih.gov/mesh), keywords and by limiting the search to human studies. The terms pancreatic cancer, pancreatic neoplasm, cutaneous metastasis and SMJN were used. The abstracts were reviewed, and articles that were not associated with to the specific topic were excluded. Duplicate references as well as repeated publications were discarded. All of the studies that were considered to be eligible were retrieved and the final selection was based on the full article. Only those patients with a confirmed pathological diagnosis, as a result of a biopsy or an autopsy of the skin lesion or pancreatic tumor, were included. Articles in which skin lesions presented with paraneoplastic syndrome associated with pancreatic cancer were excluded.

Furthermore, the reference lists were screened to identify additional eligible articles. An analysis of the published studies, concerning clinical and pathological characteristics, treatment strategies and survival outcomes, was performed. Data extraction included the following parameters: i) The age, gender and the location of the pancreatic tumors of each patient; ii) the site, number, appearance and association with any incision or surgery of the cutaneous metastatic lesions; iii) the serum tumor marker levels, histopathological types, grade and immunohistochemistry of the primary tumor; iv) lymph node and distal metastases; v) therapy, including chemotherapy, radiotherapy and surgery; and vi) survival information. In addition, the original authors were contacted for supplementary information and long-term survival data. Overall survival from the time of the initial diagnosis of the skin metastases to the date of last contact (or the date the patient succumbed to the disease) was calculated using the Kaplan-Meier method.

The literature search resulted in 62 cases of cutaneous metastasis of pancreatic cancer. The data of all 63 patients (including the current case) are summarized in Table I.

The average patient age was 62.9 years (range, 40–85 years). Males constituted a marginally greater proportion of the cohort (61.9%; 39/63), however, no significant difference was identified in patient gender.

Among the locations of the cutaneous metastases, skin lesions at non-umbilicus sites were more common than at umbilicus sites (43 vs. 18), and only two cases exhibited non-umbilicus and umbilicus metastases concurrently. The majority of the skin lesions were singular (66.7%; 42/63), particularly in patients exhibiting SMJN (90.0%; 18/20; data not shown). The predominant manifestation of the cutaneous skin metastases was a nodule or mass (73.0%; 46/63). Other manifestations included plaques, swelling or thickening (17.5%; 11/63) and one case of cellulitis. Over a quarter (28.6%; 18/63) of the skin lesions were located at local puncture or surgery locations, including surgical or biliary drainage sites (12.7%; 8/18), incision sites (3.2%; 2/18), the needle tract (11.1%, 7/18) or sites of skin transplantation due to burn (1.6%; 1/18).

Among the sites of the primary tumor, the pancreatic head or uncus accounted for 21/63 of cases (33.3%), the body for nine cases (14.3%), the tail for 16 cases (25.4%), the body and tail for eight cases (12.7%), and the majority of the pancreas in one case. Seven of the cases did not provide detailed information.

A wide range of histological subtypes were presented, with a predominance of adenocarcinoma (84.1%; 53/63), including four cases of mucin-secreting adenocarcinoma. Each of the following histological subtypes were observed in just one case of those evaluated in this report: Adenosquamous carcinoma, large-cell undifferentiated carcinoma, vasoactive intestinal polypeptide tumor, metastatic islet cell amphicrine carcinoma, mucinous cystadenocarcinoma and intraductal papillary mucinous carcinoma. The tumor histology was described without specifying the subtype in three cases.

The differentiation grade of the tumor cells was described in 32 cases and the majority of the known pathologies were graded as moderately or poorly differentiated (78.2%; 25/32). However, 31 cases did not provide detailed information regarding the differentiation grade.

Immunohistochemical analysis of CK7 was performed in 11 cases and was positive in 100% of cases, CK19 was also positive in 100% of cases (5/5), CA19-9 was positive in 91.7% of cases (11/12), CEA was positive in 75% of cases (6/8) and prostate-specific antigen was positive in 33.3% of cases (2/6). CK20 was weakly positive in one case and negative in four cases. Forty cases did not provide detailed information regarding the immunohistochemistry of the tumor cells.

Concurrent local lymph node metastasis was noted in 20/25 cases, however, the relevant information was not provided by 38 cases. Concurrent distal metastases (other than the skin) was observed in 43 cases (67.2%) including the liver (26 cases), the peritoneum (21 cases) and the lungs (11 cases). Only three cases did not develop concurrent metastases.

**Figure 4:**
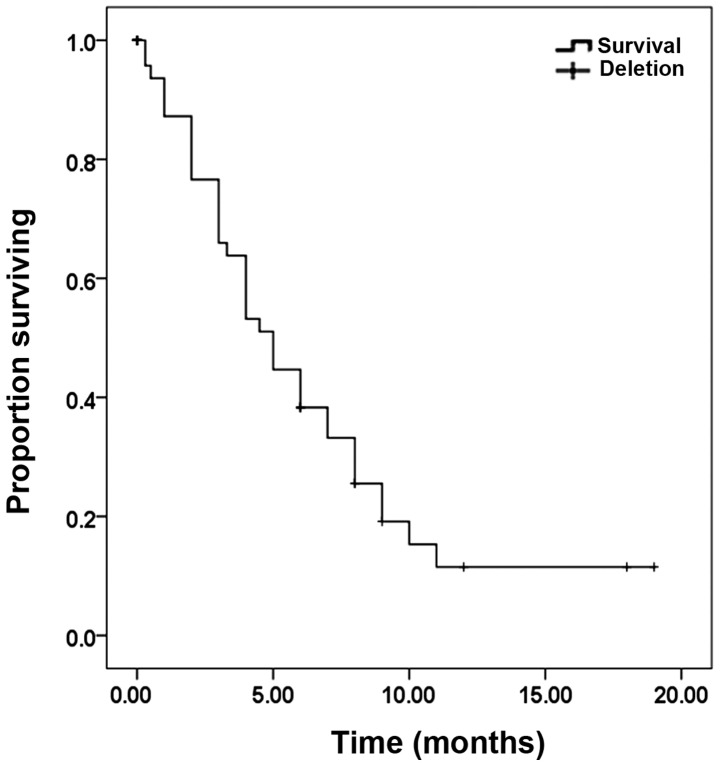
Survival curve of patients exhibiting cutaneous metastasis of pancreatic cancer. Survival time ranges from a few days to ~19 months, with a median value of five months.

**Figure 5:**
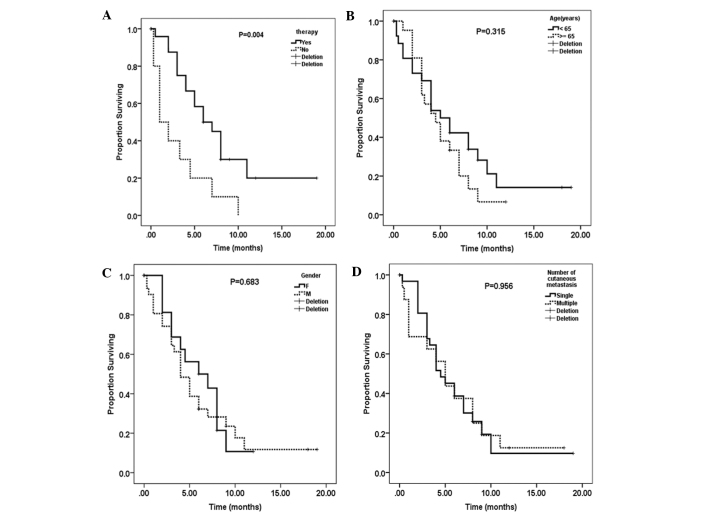
Survival curves of patients exhibiting cutaneous metastasis of pancreatic cancer grouped by therapy, age, gender and number of skin lesions. (A) Survival time of patients who underwent therapy improved significantly in comparison to patients who did not undergo therapy. However, survival time following skin metastasis was not significantly different between (B) patients <65 years and patients >65 years, (C) males and females or (D) patients with a single skin lesion and patients with multiple skin lesions. P<0.05 indicates a statistically significant difference.

Treatment consisted of chemotherapy in 20 cases, surgery in 15 cases and radiotherapy in six cases. The survival period was evaluated in 42 patients and ranged from a few days to ~19 months, with a median value of five months (Fig. 4). Therapy, including surgery, chemotherapy, radiation or a combination improved the survival time from 3.0 to 8.3 months (P=0.004; Fig. 5A). Younger patients (aged <65 years) exhibited improved survival when compared with those aged >65 years, with a mean survival time of 7.0 vs. 5.1 months, respectively; however, the difference was not identified to be significant (P=0.315; Fig. 5B). Gender and the number of skin lesions had no influence on overall patient survival (Fig. 5C and D).

## Discussion

Metastasis of pancreatic cancer is the leading cause if disease worldwide, and the pattern of metastasis commonly includes the regional lymph nodes, liver, peritoneum, lungs and brain. Uncommon sites of metastases from pancreatic cancer include the muscle, skin, heart, pleura and stomach (8). Cutaneous metastasis from pancreatic carcinoma is considered to be particularly rare (3,4). In a study of 420 patients with cutaneous metastases, Lookingbill *et al* (9) identified just two cases (0.48%) originating in the pancreas. Cubilla and Fitzgerald (10) reported that 9/119 patients (7.6%) with pancreatic cancer exhibited cutaneous metastases at autopsy. In the present report 63 cases of cutaneous metastases originating from pancreatic cancer were identified during a systematic literature review and are discussed in detail.

The review revealed that cutaneous metastatic disease from pancreatic cancer occurs in all ages, ranging from 40 to 85 years, however, more commonly occurs in older patients, with a mean age of 62.9 years and 63.5% patients aged >60 years. The gender ratio of males to females was 1.63:1 (39:24), demonstrating a male preponderance. This was consistent with a recent report by Yendluri *et al* (11), in which the male to female ratio was 1.3:1.

Horino *et al* (12) reported 49 cases of metastatic pancreatic carcinoma to the skin and revealed that skin lesions were the initial sign of pancreatic cancer in 46 cases (93.9%), with umbilical lesions being the most common metastatic location on the skin (45.5%). Miyahara *et al* (13) also reported that cutaneous metastases were present prior to the diagnosis of pancreatic cancer in 20/22 patients (90.1%). Metastatic lesions in the skin were the first symptoms of pancreatic cancer in 11 cases and the lesions were identified by physical examination in nine cases. The present review identified that skin lesions were the first sign of pancreatic cancer in 35 cases (55.6%). Concurrent physical examination identified seven cases (10.9%) and just 18 cases were identified subsequent to the diagnosis of pancreatic carcinoma.

Miyahara *et al* (13) reported that skin lesions were present in the umbilicus in 16/22 cases (72.7%) of cutaneous metastasis of pancreatic cancer. However, in the present review, a high proportion of non-umbilicus (vs. umbilicus) lesions were recorded, with a ratio of 43:18 (2.4:1).

Pancreatic cancer has the ability to metastasize to all cutaneous tissue, most frequently to the umbilicus. Non-umbilical cutaneous nodules have also been observed on the scalp (4), neck (14), temple (5), thorax, epigastric region and axilla (15,16). Local surgery is closely associated with the site of the cutaneous metastases of pancreatic cancer. Data from the present review concurred with this, identifying 18 cases (28.6%) in which the metastatic lesion was closely associated with the site of local drainage, incision, surgery, needle puncture or skin transplantation (17–23). Therefore, careful management should be taken during surgery to avoid the implantation of tumor cells that results in metastasis.

The locations of the primary pancreatic carcinoma in the present review were the head (33.3%), body (14.3%), tail (25.4%), and the body and the tail (12.7%) of the pancreas. This corresponds with a previous report by Horino *et al* (12), which identified that the locations of the primary pancreatic carcinoma were the head (32.3%), body (12.9%), tail (32.3%) and the body and the tail (19.4%) of the pancreas. Yendluri *et al* (11) stated that although 70–80% of pancreatic cancer arises in the head of the pancreas, in patients presenting with an SMJN, the majority (91%) were in the tail and body of the pancreas. The present review was consistent with this, with 76.2% of pancreatic tumors present in the tail and body of the pancreas of the SMJN patients. However, in the present report, skin lesions resulting from a primary tumor in the head of the pancreas account for a relatively smaller proportion (36.6%; 9/25) of non-umbilical cutaneous metastases from pancreatic carcinoma. Hafez (24) reviewed 17 cases of non-umbilical cutaneous metastasis from pancreatic carcinoma and identified that the site of the pancreatic tumor was commonly in the head of the pancreas (52.8%). We hypothesize that this may be due to tumors of the pancreatic head having a different metastatic route from tumors of the pancreatic tail.

The present report identified that the histological subtype of pancreatic cancer varies, although adenocarcinoma accounts for 84.1% of cases. Moderately or poorly differentiated grade tumors constitute 78.1% (25/32) of those cases with a known pathology. This is due to poorly differentiated tumor cells exhibiting a more aggressively invasive phenotype (25).

Immunohistochemistry demonstrating positivity for CK7, CK19 and CA19-9 indicated that these markers had high specificity in the diagnosis of pancreatic cancer. This is important, particularly in cases where the skin lesion is observed prior to the primary tumor. The expression of CK20 can be variable; Matros *et al* (26) used tissue specimens from 103 patients and demonstrated that CK20 expression was present in 63% of pancreatic adenocarcinoma. However, in the present review CK20 was weakly positive in one case and negative in four cases, indicating that CK20 negativity may serve as a specific diagnostic marker of pancreatic carcinoma. The case presented in the current report identified that the tumor cells were positive for CK7 and CK19, and negative for CK20, estrogen and progesterone receptors, as well as thyroid transcription factor-1. This immunophenotype was consistent with metastasis from the patient’s primary diagnosis of pancreatic adenocarcinoma.

In the present study 70% of cases exhibited elevated leveks of CA19-9 and CEA. However, in a study by Gui *et al* (27), the proportion of patients with elevated CA19-9 and CEA levels was 80.3%.

When cutaneous metastases are present, pancreatic cancer is usually widely disseminated. Takeuchi *et al* (16) reported the involvement of other organs at the time of diagnosis of the skin metastases in eight out of nine patients. Horino *et al* (12) reviewed 49 reported cases of pancreatic metastasis and found that 90.3% of the cases exhibited multiple organ metastases or peritoneal seeding. The present review demonstrated that in 43/46 (93.5%) cases that were evaluated, distal metastasis had occurred. The majority of distal metastases occurred in the liver, followed by the peritoneum and the lungs, which is consistent with the above-mentioned reports.

Various theories of cutaneous metastasis have been proposed, however, no specific mechanism has been elucidated. These theories include the soil and seed hypothesis, direct invasion, lymphatic or hematogenous dissemination and the chemotaxis hypothesis (11,12,28,29). Tumor seeding during resection is a feared complication as recurrence within the peritoneal cavity commonly occurs following resection with curative intent. Consistent with this theory, the present review identified 18 cases in which tumor seeding was associated with trauma.

According to Yendluri *et al* (11), the average survival time for SMJN patients was less than four months, which is the time scale expected for stage IV pancreatic cancer. Takeuchi *et al* (16) reported that seven out of nine cases of skin metastasis from pancreatic cancer succumbed within seven months of the diagnosis. The findings of the present report demonstrated that survival time ranged from a few days to ~19 months with a median value of five months, which is consistent with the above-mentioned reports. Therapy, including surgery, chemotherapy, radiation or a combination improved the survival time from 3.0 to 8.3 months (P=0.004). This significant difference may be explained by certain patients having a low Karnofsky score (30), therefore, not being physically able to undergo curative surgery. Younger patients (aged <65 years) had a longer survival time when compared with those patients aged >65 years, with a mean survival time of 7.0 vs. 5.1 months, respectively; however, the difference was not significant (P=0.315). Only 63 patients were evaluated in the present review and a larger sample size may have led to a significant difference. By contrast, there was no difference in survival between males and females or between patients with a single skin lesion and multiple skin lesions.

In conclusion, pancreatic cancer rarely presents as cutaneous metastases, however, this possibility should be considered in the differential diagnosis, particularly when the malignant skin lesion is of unknown origin. Immunohistochemical staining with a specific antibody (for example CK19 or CK7) may aid in the elucidation of the origin of the underlying tumor, which may guide further management of the treatment strategy. Although the patients exhibited stage IV pancreatic cancer with cutaneous metastasis, they appeared to demonstrate improved outcomes as a result of treatment with a combination of surgery, radiotherapy and chemotherapy.

## Figures and Tables

**Table I:** Clinical characteristics of patients with cutaneous metastasis from pancreatic cancer.

Characteristic	Patients, n (%)
Age, years	
≤60	23 (36.5)
60–80	34 (54)
>80	6 (9.5)
Gender	
Male	39 (61.9)
Female	24 (38.1)
Location	
Umbilicus only	18 (28.6)
Non-umbilicus only	43 (68.3)
Umbilicus and non-umbilicus concurrently	2 (3.1)
Skin metastasis and pancreatic cancer	
Skin lesion first	35 (55.6)
Pancreatic cancer first	18 (28.6)
Concurrently	7 (11.1)
No details	3 (4.8)
Appearance	
Nodule or mass	46 (73.0)
Plaque, swelling or thickening	11 (17.5)
Cellulitis	1 (1.6)
No details	5 (7.9)
Lesions, n	
Single	42 (66.7)
Multiple	21 (33.3)
Association with local surgery	
Yes	
Drainage site	8 (12.7)
Incision site	2 (3.2)
Needle tract	7 (11.1)
Transplanted skin	1 (1.6)
No	45 (71.4)
Primary tumor site	
Head or uncus	21 (33.3)
Body	9 (14.3)
Tail	16 (25.4)
Body and tail	8 (12.7)
Majority of the pancreas	1 (1.6)
No details	7 (11.1)
Histology	
Adenocarcinoma	53 (84.1)
Mucin-secreting adenocarcinoma	4 (6.3)
Adenosquamous cell carcinoma	1 (1.6)
Large cell undifferentiated carcinoma	1 (1.6)
Vasoactive intestinal polypeptide tumor	1 (1.6)
Metastatic islet cell amphicrine carcinoma	1 (1.6)
Mucinous cystadenocarcinoma	1 (1.6)
Intraductal papillary mucinous carcinoma	1 (1.6)
Lobular panniculitis	1 (1.6)
No details	3 (4.7)
Grade (32 cases available)	
Well-differentiated	7 (21.8)
Moderately or poorly differentiated	25 (78.1)
Immunochemistry	
CK7-positive	11/11 (100.0)
CK19-positive	5/5 (100.0)
CA19-9-positive	11/12 (91.7)
CEA-positive	6/8 (75.0)
CK20-negative	4/5 (80.0)
PSA-positive	2/6 (33.3)
Concurrent metastasis	
Lymphoma metastasis (25 cases available)	
Yes	20 (80.0)
No	5 (20.0)
Distal metastasis (43 cases available)	
Liver	26 (60.5)
Peritoneum	21 (48.8)
Lung	11 (25.6)
No distal metastasis	3 (7.0)
Therapy (34 cases available)	
Chemotherapy	20 (58.8)
Surgery	15 (44.1)
Radiation	6 (17.6)
No therapy	10 (29.4)
Survival time after skin metastasis (47 cases available)	
<6 months	25 (53.2)
≥6 months	22 (46.8)

CK, cytokeratin; CA, carbohydrate antigen; CEA, carcinoembryonic antigen; PSA, prostate-specific antigen.
